# Supplementary material for: A Cell Cycle‐Aware Network for Data Integration and Label Transferring of Single‐Cell RNA‐Seq and ATAC‐Seq
Source: Adv Sci (Weinh). 2024 Jun 17;11(31):2401815. doi: 10.1002/advs.202401815 (PMC11336957; doi:10.1002/advs.202401815)
Supplement: Supplementary file 1 — Supporting Information [file ADVS-11-2401815-s001.pdf]

ADVANCED SCIENCE
Open Access

Supporting Information

for *Adv. Sci.*, DOI 10.1002/advs.202401815

A Cell Cycle-Aware Network for Data Integration and Label Transferring of Single-Cell RNA-Seq and ATAC-Seq

*Jiajia Liu, Jian Ma, Jianguo Wen and Xiaobo Zhou**

Supporting Information

For

A Cell Cycle-aware Network for Data Integration and Label Transferring of Single-cell RNA-seq and ATAC-seq

Jiajia Liu, Jian Ma, Jianguo Wen, Xiaobo Zhou[*]

Figure S1

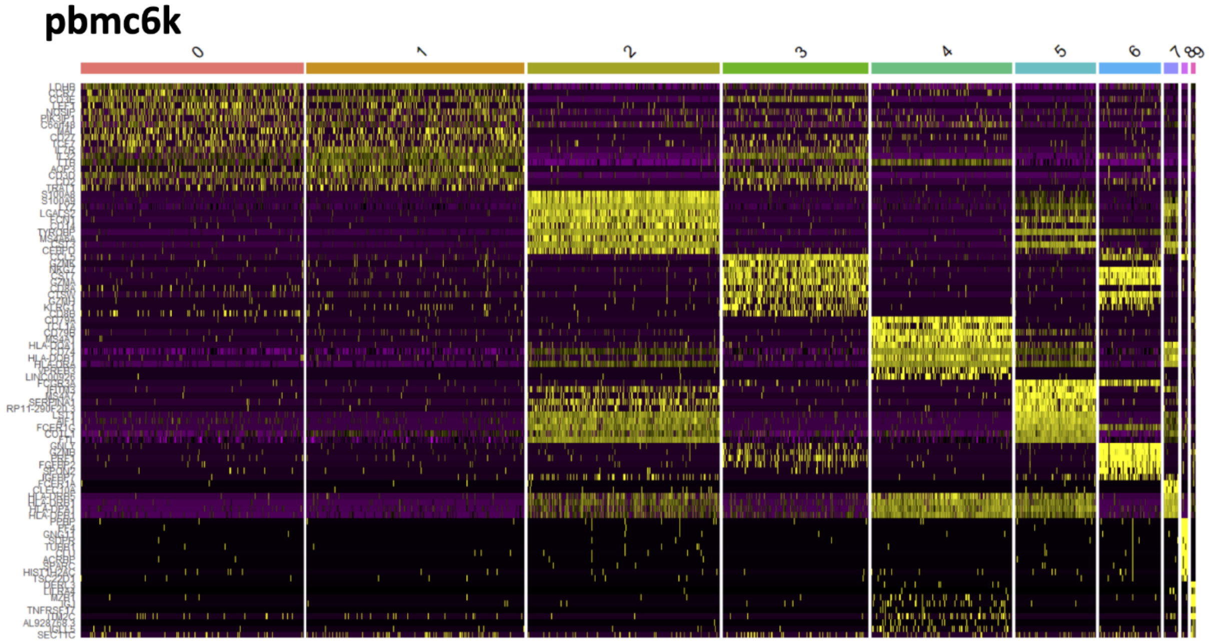

cell types	markers	clusters
monocytes	CD14+	2
dendritic cells	FCER1A+	7
B cells	MS4A1+	4
T cells	CD3D+	0,1,3
megakaryocytes	PPBP+	8
natural killer (NK) cells	CD3D-/GNLY+	6
FCGR3A+ Mono	FCGR3A+/MS4A7+	5
Plasma cell (effector B cells)	DERL3	9

finalize →

cell types	markers	clusters
monocytes	CD14+	2,5
dendritic cells	FCER1A+	7
B cells	MS4A1+	4,9
T cells	CD3D+	0,1,3
megakaryocytes	PPBP+	8
natural killer (NK) cells	CD3D-/GNLY+	6

Figure S1. Cell type annotation of pbmc_6k scRNA-sea data using canonical markers.

Figure S2

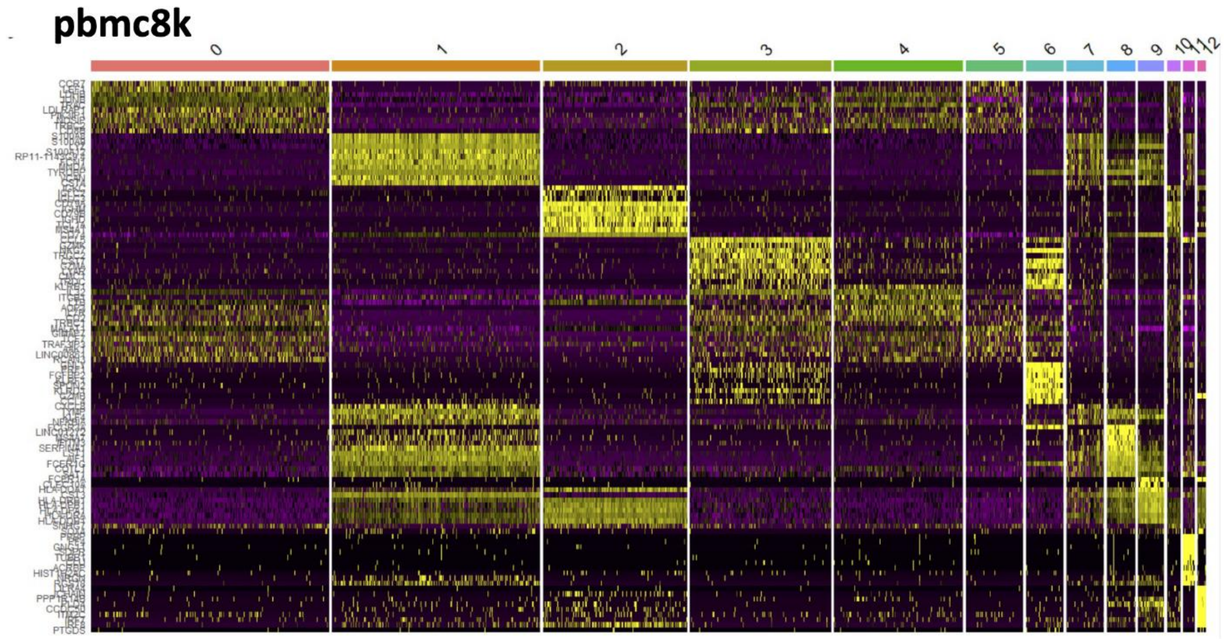

cell types	markers	clusters
monocytes	CD14+	1
dendritic cells	FCER1A+	9,12
B cells	MS4A1+	2
T cells	CD3D+	0,3,4,5,10
megakaryocytes	PPBP+	11
natural killer (NK) cells	CD3D-/GNLY+	6
FCGR3A+ Mono	FCGR3A+/MS4A7+	8
CD14+ Mono	LYZ+	7

finalize

cell types	markers	clusters
monocytes	CD14+	1,7,8
dendritic cells	FCER1A+	9,12
B cells	MS4A1+	2
T cells	CD3D+	0,3,4,5,10
megakaryocytes	PPBP+	11
natural killer (NK) cells	CD3D-/GNLY+	6

Figure S2. Cell type annotation of pbmc_8k scRNA-sea data using canonical markers.

Figure S3

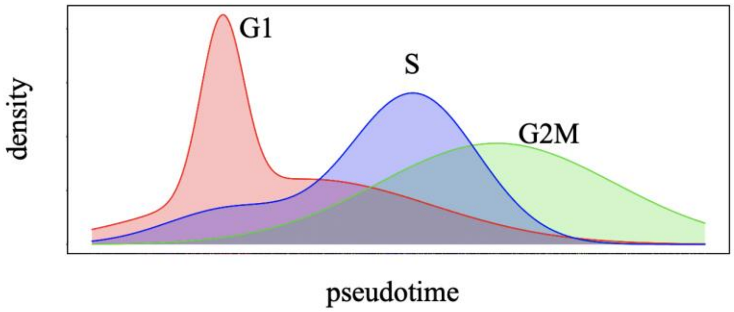

Figure S3. The density plot of pseudotime for each cell cycle phase, peaks are estimated using a Gaussian Mixture Model (GMM) with three components.

Figure S4

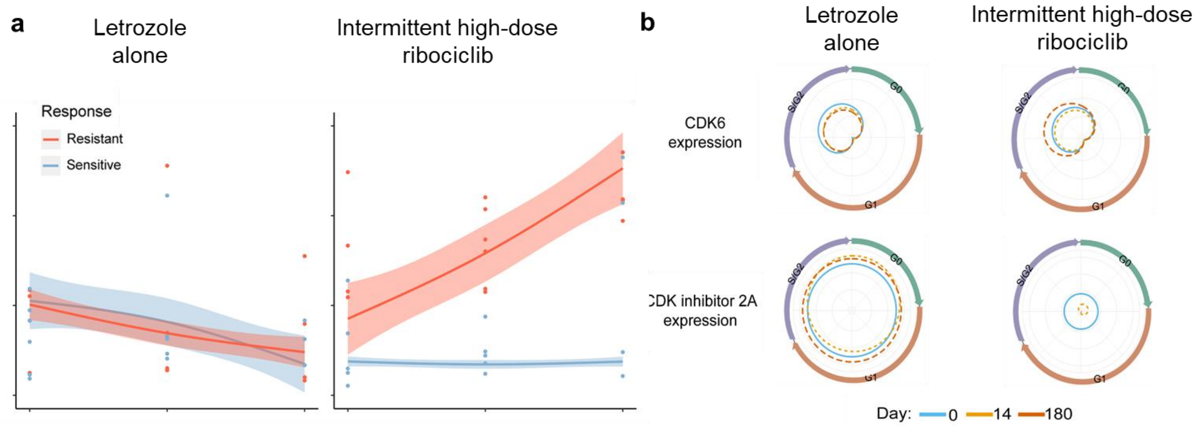

Figure S4. Analysis of cell cycle dysregulation identified by CCAN. a, Changes in the proportion of cells at different stages of the cell cycle (S/G2 phase) in patient tumor samples treated with Letrozole alone and intermittent high-dose Ribociclib. Blue represents patients sensitive to treatment, and red represents patients resistant to treatment, with changes in cell proportions over time. b, Changes in the expression of CDK6 and CDKN2A along the cell cycle before, during, and after the treatment. Colored lines show the expected gene expression in cells throughout the cell cycle before (blue), during (orange), and after (red) treatment. The distance from the center of the circle indicates gene expression at a particular point in the cell cycle.

Figure S5

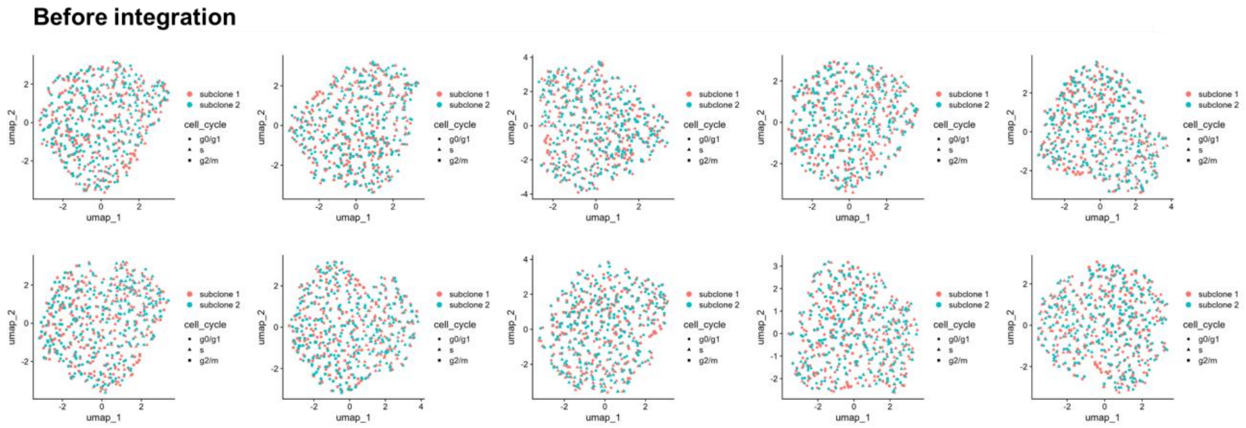

Figure S5. UMAP visualizations of 10 simulated datasets before removing the cell cycle effect.

Figure S6

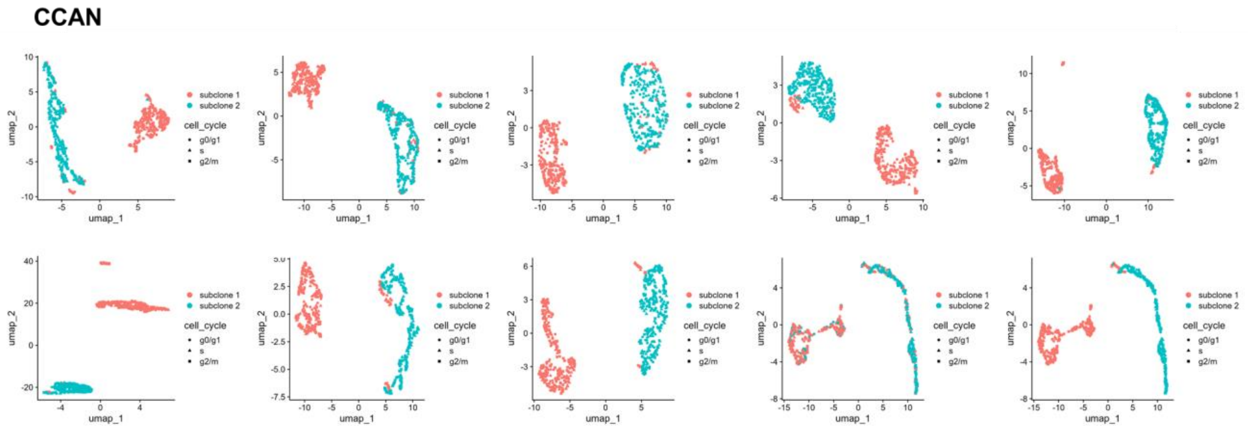

Figure S6. UMAP visualizations of 10 simulated datasets after removing the cell cycle effect using CCAN.

Figure S7

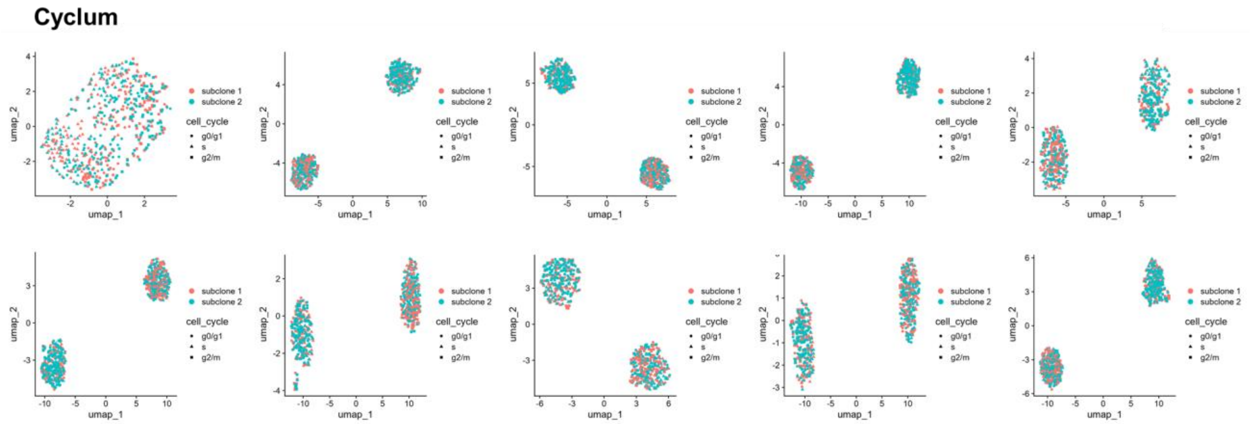

Figure S7. UMAP visualizations of 10 simulated datasets after removing the cell cycle effect using Cyclum.

Figure S8

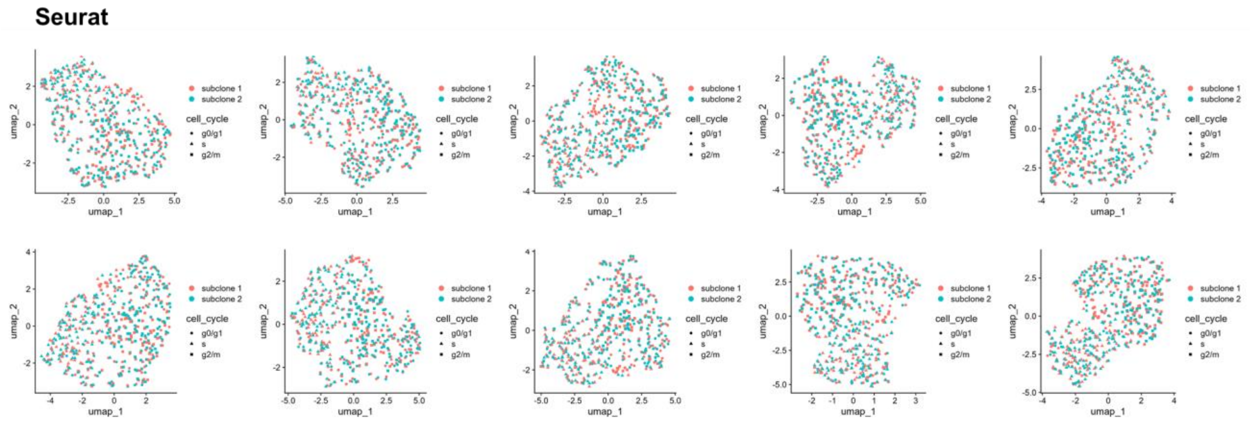

Figure S8. UMAP visualizations of 10 simulated datasets after removing the cell cycle effect using Seurat.

Figure S9

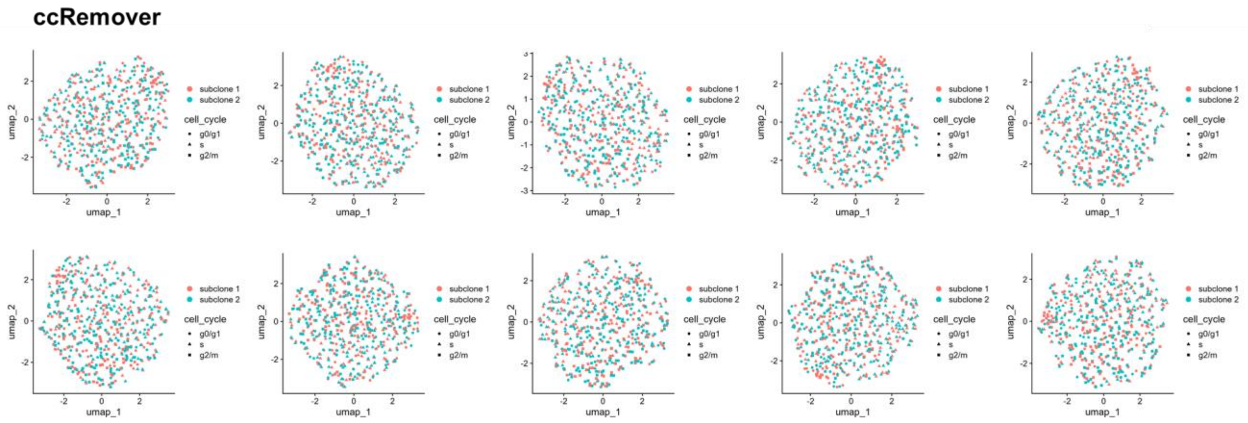

Figure S9. UMAP visualizations of 10 simulated datasets after removing the cell cycle effect using ccRemover.

Figure S10

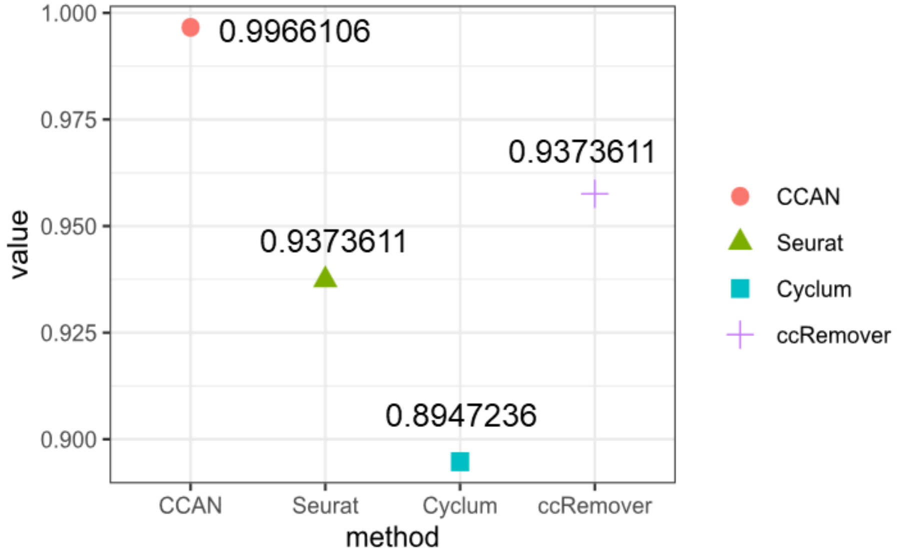

Figure S10. The separability of PBMC scRNA-seq dataset after removing cell cycle effect using CCAN, Seurat, Cyclum and ccRemover.

Figure S11

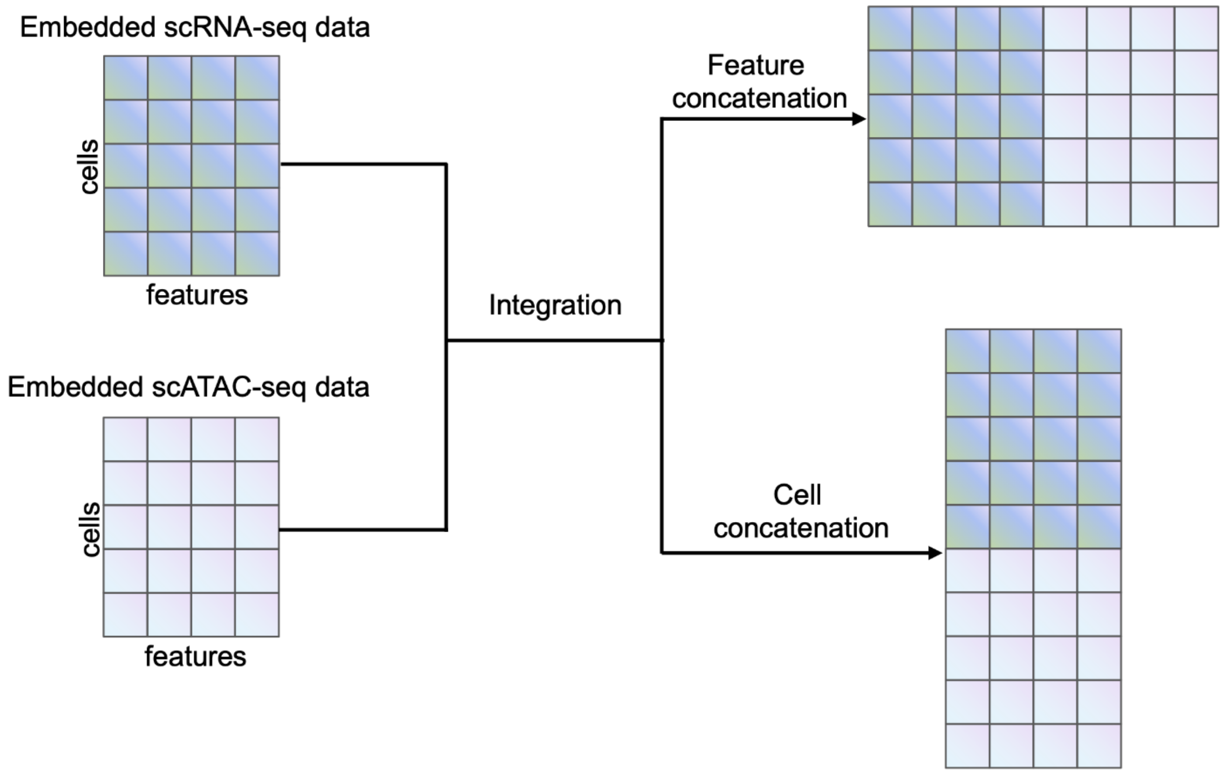

Figure S11. Illustration of feature concatenation and cell concatenation for paired data when integration.

Figure S12

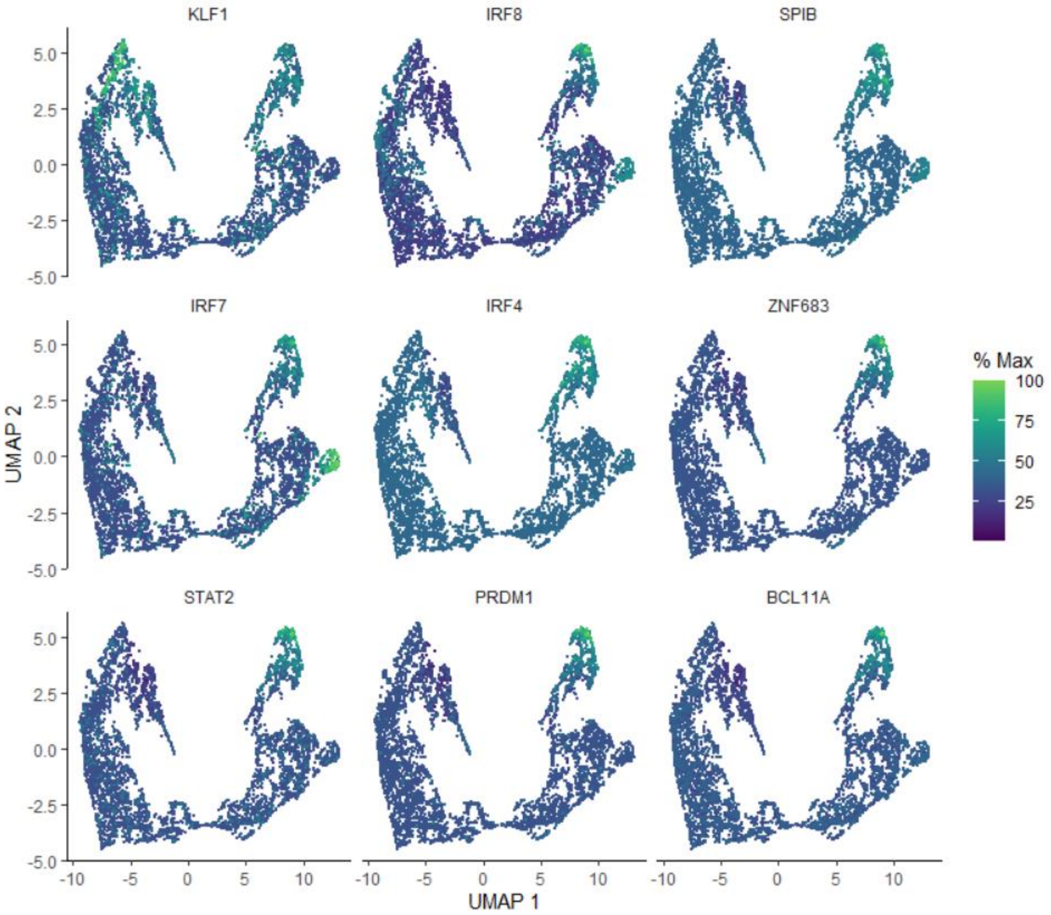

Figure S12. UMAP visualization of integrated scRNA-seq data and scATAC-seq data colored by gene expression of nine highly-correlated genes.

Figure S13

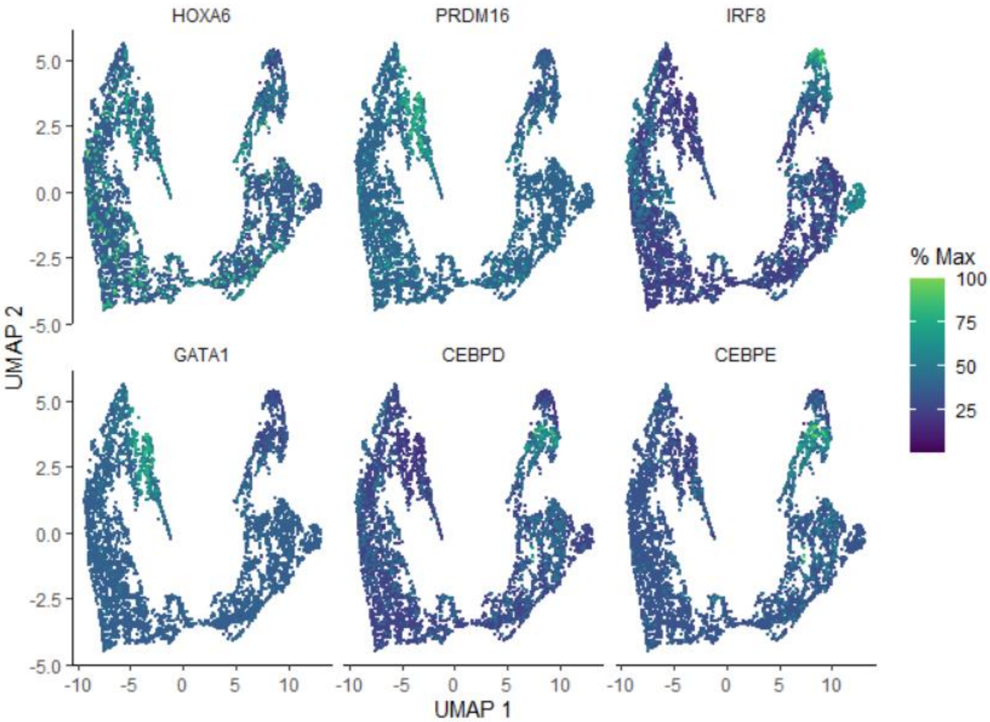

Figure S13. UMAP visualization of integrated scRNA-seq data and scATAC-seq data colored by gene expression of differentially expressed genes identified by DeSeq2.

Table S1

NO.	Abbreviation	Tissue	Platform	Cell type labeled	Number of cell types	Reference	Data Sources	Number of cells in source domain	Number of genes in source domain	Number of cells in target domain	Number of genes in target domain
1	harmony_pbmc	human peripheral blood mononuclear cells (PBMCs)	assayed on the Chromium 10X platform but prepared with different protocols: 3′ end v1 (3pV1) and 3′ end v2 (3pV2).	annotate using canonical cell type marker genes	6	Korsunsky I, Millard N, Fan J, et al. Fast, sensitive and accurate integration of single-cell data with Harmony[J]. Nature methods, 2019, 16(12): 1289-1296.	https://support.10xgenomics.com/single-cell-gene-expression/datasets/1.1.0/pbmc6k https://support.10xgenomics.com/single-cell-gene-expression/datasets/2.1.0/pbmc8k	5,356	13,640	8,806	13,640
2	snare_adbrain	adult mouse brain cerebral cortex	SNARE-seq	annotate by transferring labels from an existing scRNA-seq dataset produced by the Allen Institute	13	Chen S, Lake BB, Zhang K. High-throughput sequencing of the transcriptome and chromatin accessibility in the same cell. Nat Biotechnol 2019 Dec;37(12):1452-1457. PMID: 31611697	GSE126074	8,055	2,088	8,055	2,088
3	unpaired scRNA-seq and scATAC-seq data	human PBMC	10x Genomics scATAC-seq v1.1 (ASAP-seq) / scRNA-seq v3.1 (CITE-seq), control group	labeled	7	Mimitou EP, Lareau CA, Chen KY, Zorzetto-Fernandes AL et al. Scalable, multimodal profiling of chromatin accessibility, gene expression and protein levels in single cells. Nat Biotechnol 2021 Oct;39(10):1246-1258. PMID: 34083792	GSE156478	4,644	17,441	4,157	17,441
4	single-cell differentiated data	Homo sapiens bone marrow/human hematopoietic differentiation	10X v2 sequencing	labeled	6	Setty M, Kiseliovas V, Levine J, et al. Characterization of cell fate probabilities in single-cell data with Palantir[J]. Nature biotechnology, 2019, 37(4): 451-460. Buenrostro JD, Corces MR, Lareau CA, Wu B et al. Integrated Single-Cell Analysis Maps the Continuous Regulatory Landscape of Human Hematopoietic Differentiation. Cell 2018 May 31;173(6):1535-1548.e16. PMID: 29706549	https://data.humancellatlas.org/explore/projects/091cf39b-01bc-42e5-9437-f419a66c8a45/project-matrices GSE96772	4,135	1,015	1,594	1,015
5	mESCs data	mouse embryonic stem cells	HighSeq 2000 sequencing system	No, but cell cycle labeld		Buettner, Florian, et al. "Computational analysis of cell-to-cell heterogeneity in single-cell RNA-sequencing data reveals hidden subpopulations of cells." Nature biotechnology 33.2 (2015): 155-160.	https://www.ebi.ac.uk/arrayexpress/experiments/E-MTAB-2805/	288 cells			
6	Breast cancer single-cell dataset	human	10x technology	No		Griffiths, Jason I., et al. "Serial single-cell genomics reveals convergent subclonal evolution of resistance as patients with early-stage breast cancer progress on endocrine plus CDK4/6 therapy." Nature cancer 2.6 (2021): 658-671.	GSE158724	176,644 cells			

Table S2

NO.	Methods	scRNA-seq	Paired	Unpaired	Input format
1	Harmony	√			gene expression matrix
2	Conos	√			gene expression matrix
3	Scanorama	√			gene expression matrix
4	BBKNN	√			gene expression matrix
5	Seurat v4	√	√	√	gene expression + ATAC gene activity matrix
6	online iNMF	√	√	√	gene expression count + ATAC gene activity count (same feature numbers)
7	scMVP		√		top DEG scRNA matrix + TF-IDF normalized/binary scATAC matrix
8	scAI		√		scRNA matrix + sparse/binary scATAC matrix
9	scMVAE		√		the raw count data of scRNA-seq + scATAC data (gene activity format). Row indicates variable (genes and loci), and column indicates sample (cell).
10	scJoint		√	√	gene expression + ATAC gene activity score matrix
11	GLUE		√	√	gene expression profile + peak matrix with AnnData format as input
12	SCALEX		√	√	sparse matrix of gene expression and gene activity

Text S1: Details of multiclass classification metrics

We define TP, TN, FP, FN as follows

	Predicted Class		
Actual Class		Class=Yes	Class= No
	Class=Yes	TP	FN
	Class=No	FP	TN

TP is the number of true positives, TN is the number of true negatives, FP is the number of false positives, and FN is the number of false negatives. Details of the clustering metrics used in our study are shown as follows

1. Rand Index (RI): the Rand index represents the frequency of occurrence of agreements over the total pairs, it can also represent as a measure of the percentage of correct decisions made by the algorithm. It can be computed using the following formula

$$RI = \frac{TP + TN}{TP + FP + FN + TN}$$

   Properties: the Rand index has a value between 0 and 1, with 0 indicating that the two clusters do not agree on any pair of points and 1 indicating that the data clusters are exactly the same [1]. The disadvantage of the Rand index is that it cannot handle randomly assigned clusters. As the number of clusters increases, the Rand index of the randomly assigned clusters will increase, which should be close to zero. Therefore, we also considered the Adjusted Rand Index.

2. Adjusted Rand Index (ARI) [2]: the adjusted Rand Index is a modified version of the Rand Index [1, 3, 4]. The adjusted Rand index corrects for chance and will give a baseline for the randomly assigned clustering problem in the Rand Index. It is calculated as

$$ARI = \frac{\sum_{ij}\binom{n_{ij}}{2} - [\sum_i \binom{a_i}{2} \sum_j \binom{b_j}{2}]/\binom{n}{2}}{\frac{1}{2}\left[\sum_i \binom{a_i}{2} + \sum_j \binom{b_j}{2}\right] - [\sum_i \binom{a_i}{2} \sum_j \binom{b_j}{2}]/\binom{n}{2}}$$

   Properties: the bounded range of the Adjusted Rand Index is [-1, 1]: negative values are bad (independent labels), similar clustering results have a positive ARI, 1.0 is the perfect match score. No assumption is made on the cluster structures [5].

3. Normalization Mutual Information (NMI): Normalized Mutual Information (NMI) is a normalization of the Mutual Information (MI) score to scale the results between 0 (no mutual information) and 1 (perfect correlation). Mutual Information tells us the reduction in the entropy of class labels that we get if we know the cluster labels. It is calculated as

$$NMI = \frac{H(X) + H(Y) - H(X,Y)}{(H(X) + H(Y))/2}$$

where $H(X)/H(Y)$ is the entropy of the random variable X/Y associated with two partitions, whereas $H(X,Y)$ is the joint entropy [6].

Properties: It can be used to compare two clustering results that have different number of clusters

4. Accuracy: Accuracy in classification problems is the number of correct predictions made by the model over the ground truth. It is calculated as

$$Accuracy = \frac{TP + TN}{N}$$

N is the number of samples.

Properties: Accuracy is a good measure when the target variable classes in the data are nearly balanced. Accuracy is equal to Rand index in the binary classification, but they are different in multiclass classification in CCPE.

5. Precision: the precision in the binary classification is defined as the number of true positives TP over the number of true positives TP plus the number of false positives FP as the following

$$Precision = \frac{TP}{TP + FP}$$

In the classification of CPPE, there are three clusters representing G1, S and G2/M phases. We calculate the precision for each phase and get three precision values. The Macro-Precision [7] used in CCPE is calculated as

$$Macro - Precision = \frac{Precision_{G1} + Precision_{S} + Precision_{G2/M}}{3}$$

Properties: the precision is a measure of result relevancy and it is useful to measure the prediction of multiple classes.

6. Recall: the recall in the binary classification is defined as the number of true positives TP over the number of true positives TP plus the number of false negatives FP as the following

$$Recall = \frac{TP}{TP + FN}$$

In the classification of CPPE, there are three clusters representing G1, S and G2/M phases. We calculate the recall for each phase and get three recall values. The Macro-Recall [7] used in CCPE is calculated as

$$Macro - Recall = \frac{Recall_{G1} + Recall_{S} + Recall_{G2/M}}{3}$$

Properties: the recall is a measure of how many truly relevant results are returned, also known as true positive rate.

7. Fscore: the Fscore in the binary classification is the harmonic mean of precision and recall and is calculated as

$$Fscore = 2 \cdot \frac{Precision \cdot Recall}{Precision \times Recall}$$

Macro-Fscore [7] used in CCPE for multiclass classification is calculated as

$$Macro - Fscore = 2 \cdot \frac{Macro - Precision \cdot Macro - Recall}{Macro - Precision \times Macro - Recall}$$

Properties: harmonic mean is kind of an average when precision and recall are equal.

Supporting References

1. Rand, W.M., *Objective criteria for the evaluation of clustering methods.* Journal of the American Statistical association, 1971. **66**(336): p. 846-850.
2. Mao, Q., et al. *Dimensionality reduction via graph structure learning*. in *Proceedings of the 21th ACM SIGKDD International Conference on Knowledge Discovery and Data Mining*. 2015.
3. Hubert, L. and P. Arabie, *Comparing partitions.* Journal of classification, 1985. **2**(1): p. 193-218.
4. Vinh, N.X., J. Epps, and J. Bailey, *Information theoretic measures for clusterings comparison: Variants, properties, normalization and correction for chance.* The Journal of Machine Learning Research, 2010. **11**: p. 2837-2854.
5. Yeung, K.Y. and W.L. Ruzzo, *An empirical study on principal component analysis for clustering gene expression data.* Bioinformatics, 2001. **17**(9): p. 763-774.
6. Lancichinetti, A., S. Fortunato, and J. Kertész, *Detecting the overlapping and hierarchical community structure in complex networks.* New journal of physics, 2009. **11**(3): p. 033015.
7. Sokolova, M. and G. Lapalme, *A systematic analysis of performance measures for classification tasks.* Information Processing & Management, 2009. **45**(4): p. 427-437.
